# The Cardiac Autoantibody Fingerprint of Chagas Cardiomyopathy: Towards Accessible Risk Stratification

**DOI:** 10.1016/j.jacbts.2026.101733

**Published:** 2026-09-22

**Authors:** Susanne Sattler

**Affiliations:** Division of Pharmacology, Otto-Loewi Research Center / Department of Cardiology, LKH Univ. Klinikum Graz, Medical University of Graz, Graz, Austria

**Keywords:** autoantibodies, biomarkers, Chagas disease, cardiomyopathy, disease severity

**Figure:**
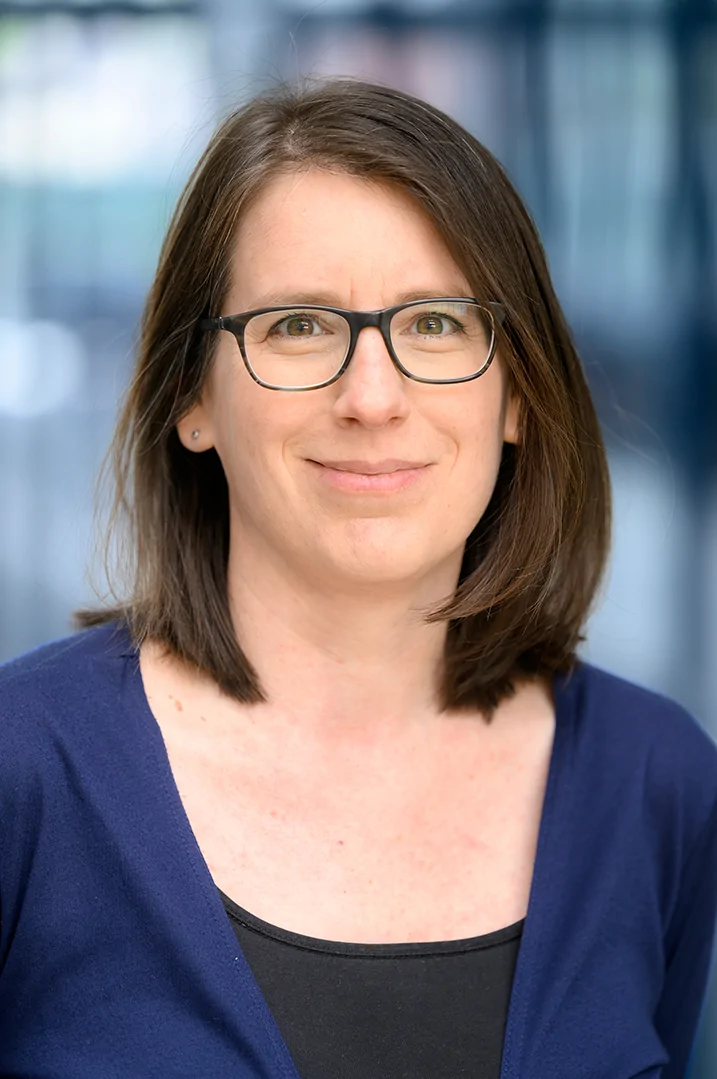

Chagas disease, caused by the protozoan pathogen *Trypanosoma cruzi*, is the most common parasitic disease in the Western Hemisphere with nearly 6 million people affected.^1^ Chagas disease is often described as a silent disease because many patients are not aware they carry a chronic *T cruzi* infection until, sometimes after decades, one-third develop Chagas cardiomyopathy (ChCM), which may progress to arrhythmia, heart failure, and sudden death. Although echocardiography is indispensable for diagnosis, it largely identifies cardiac involvement once established.^2^ A low-cost blood biomarker capable of identifying ChCM, or more importantly, predicting disease progression, would therefore address a substantial unmet need, particularly in regions where access to advanced imaging technology and specialist follow-up remains a hurdle to effective treatment (Figure 1).

**Figure 1:**
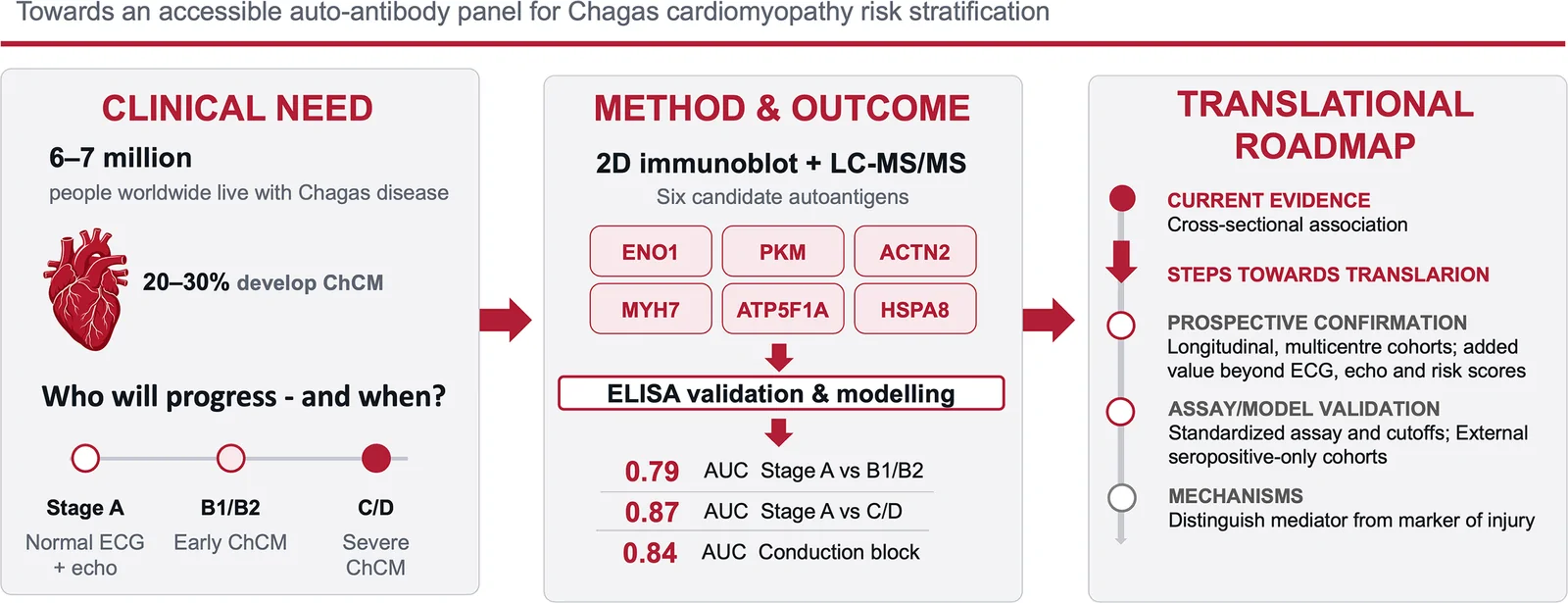
Making Chagas Cardiomyopathy Risk Visible Findings presented in Ribeiro et al^5^ (central box) in the context of clinical need (left box) and translational opportunities (right box). 2D = 2-dimensional; ACTN2 = actinin alpha 2; ATP5F1A = ATP synthase F1 subunit alpha; AUC = area under the curve; ChCM = Chagas cardiomyopathy; ECG = electrocardiogram; ELISA = enzyme-linked immunosorbent assay; ENO1 = enolase 1; HSPA8 = heat shock protein family A member 8; LC-MS/MS = liquid chromatography-tandem mass spectrometry; MYH7 = myosin heavy chain 7; PKM = pyruvate kinase M1/2.

The underlying infectious disease biology of ChCM supports looking at the immune response for such a biomarker. ChCM is driven principally by persistent infection and dysregulated host immune responses, with microvascular dysfunction and autonomic denervation acting as additional contributors to myocardial injury, fibrosis, and electrical instability.^3^ Autoantibodies against cardiac myosin, β1-adrenergic and M2-muscarinic receptors, and oxidatively modified myocardial proteins have long been associated with ChCM development, indicating a role for autoimmunity.^4^

In this issue of *JACC-BTS*, Ribeiro et al^5^ used an elegant strategy to identify a serologic autoantibody fingerprint associated with ChCM severity. This study is thus part of a broader effort to translate antiheart autoantibodies from correlative observations into clinically useful information. Autoantibodies targeting β1-adrenergic and M2-muscarinic receptors, cardiac myosin, troponin I, and other myocardial antigens have been described in myocarditis, dilated cardiomyopathy, ischemic injury, and heart failure.^6^ A consensus is therefore emerging that sees autoimmunity as a common aspect of heart failure.^7^ Yet clinical adoption has been limited by patchy data, missing standardized assays, and lack of disease specificity. In addition, uncertainty remains over whether autoantibodies precede tissue injury and might thus be causative, or arise as bystanders through passive antigen release and epitope spreading. To complicate matters, both may happen in parallel, as different autoantibodies will have a variety of different effector mechanisms depending on isotype and specificity. However, circulating antibodies can be very useful biomarkers, because of their stability and ease of detection, even if only bystanders without a causative pathogenic role. By testing a multiantibody signature, the present study moves beyond individual antibodies towards a “humoral fingerprint” which may better capture the heterogeneous immune processes accompanying cardiac injury.

Ribeiro et al^5^ investigated a cohort of 62 individuals with serologically confirmed Chagas disease ranging from stage A (asymptomatic), stages B1/B2 to stages C/D (24% NYHA functional class IV), together with 61 healthy controls or other heart disease patients. Initially, sera from 4 patients with established ChCM were screened against human ventricular protein by 2D electrophoresis and immunoblotting, followed by mass spectrometry. Six candidate proteins were identified to carry antigenic peptides: α-enolase, pyruvate kinase M, α-actinin-2, β-myosin heavy chain, ATP synthase F1 subunit α, and heat shock cognate 71-kDa protein, and were evaluated by enzyme-linked immunoadsorbent assay (ELISA). The combined antibody signature discriminated stage A from stages B1/B2 and C/D as well as between patients with and without conduction block.

This study makes several notable contributions to the field. It moves beyond individual autoantibodies and combines discovery proteomics with immunoblot and ELISA validation. The identified targets form a biologically coherent pattern involving myocardial energetics and sarcomeric integrity. α-enolase autoantibodies appeared most informative in separating stage A from early ChCM, whereas α-actinin-2 autoantibodies showed the strongest association with advanced disease. Comparison with patients with Brugada syndrome, arrhythmogenic right ventricular cardiomyopathy, and ischemic heart failure is an important attempt to distinguish a Chagas-associated signature from other cardiac injury. Finally, validation by standard ELISA, depending on neither advanced imaging nor mass spectrometry, could ultimately have particular value in endemic and resource-constrained settings. A few steps are still necessary towards this end.

(1) Establish temporal validity. In the current study, samples were collected after disease stages, and conduction phenotypes were already apparent. Longitudinal follow-up of patients in stages A and B will be essential to determine whether the antibody signature precedes clinical cardiac dysfunction. This will distinguish a marker of established myocardial injury from one that genuinely predicts progression. Such studies should also test if the proposed antibody biomarker panel adds benefit beyond electrocardiogram, echocardiography, natriuretic peptides, and established clinical risk scores such as the Rassi score,^8^ and whether serial antibody trajectories are more informative than a single measurement.
(2) Assay development. Common calibrators, transferable units, standardized sample handling, and demonstration of interassay and interlaboratory reproducibility will be needed to convert ELISA optical-density readouts into clinically useful thresholds. Expanding antigen discovery across early, indeterminate, and advanced ChCM, as well as appropriate non-Chagas disease controls, may also reveal whether a smaller, more parsimonious panel can retain performance while improving affordability and robustness.
(3) Refinement of the analytical model in larger and more diverse cohorts. Developing and validating the model exclusively among *T cruzi*-seropositive individuals would determine whether it truly identifies cardiomyopathy rather than infection itself, because the current non-ChCM group also included a large cohort of healthy participants and patients with non-Chagas cardiac disease. The evaluation of several machine-learning algorithms is a useful exploratory step, but independent external validation remains the decisive benchmark. Multicenter cohorts representing different endemic regions could provide more precise estimates of performance, address variation related to age, sex, ancestry, parasite strain, and treatment history.^9^

However, beyond a stepping stone towards clinical translation of a new ChCM biomarker panel, this study also provides the opportunity to connect biomarker discovery with underlying mechanistic research. Because the identified targets are predominantly intracellular structural and metabolic proteins, future studies could determine whether the antibodies are in fact active mediators instead of simply passive messengers of cardiomyocyte injury. Affinity-purified antibodies could be tested for binding to human myocardium and intact or injured cardiomyocytes, followed by measurements of viability and function. Relating these responses to parasite persistence, systemic inflammation, and myocardial fibrosis could provide further valuable biological resolution.

In summary, if the proposed autoantibody signature proves temporally predictive, externally reproducible, and additive to conventional testing, cardiac autoantibody-based prognostic assays could help transform the long silent interval of Chagas disease into an opportunity for targeted surveillance and earlier intervention.

## Funding Support and Author Disclosures

The authors have reported that they have no relationships relevant to the contents of this paper to disclose.
